# Association between serum NLRP3 and malignant brain edema in patients with acute ischemic stroke

**DOI:** 10.1186/s12883-021-02369-4

**Published:** 2021-09-07

**Authors:** Yanan Wang, Hexiao Huang, Weihong He, Shihong Zhang, Ming Liu, Simiao Wu

**Affiliations:** 1 Department of Neurology, West China Hospital, Sichuan University, 610041 Chengdu, China; 2 West China School of Medicine, Sichuan University, 610041 Chengdu, China; 3 Department of Physiology, West China School of Basic Medical Sciences and Forensic Medicine, Sichuan University, 610041 Chengdu, China

**Keywords:** NLRP3, Malignant brain edema, Acute ischemic stroke, Biomarkers

## Abstract

**Background:**

We aimed to explore the association of serum level of the nucleotide-binding oligomerization domain-like receptor family pyrin domain-containing 3 (NLRP3) and its related inflammatory biomarkers (hypoxia inducible factor-1α, cathepsin B, caspase-1 and matrix metalloproteinase-9) with malignant brain edema (MBE) in patients with acute ischemic stroke.

**Methods:**

We prospectively enrolled patients with acute ischemic stroke admitted < 24 h from onset of symptoms. Brain CT was performed on admission and blood samples were collected. Repeated brain CT/MRI was performed < 7 days of admission to identify the presence of MBE, defined as neurological deterioration with imaging signs of midline shift or compressed basal cisterns. Logistic regression analysis was performed to assess the association between inflammatory biomarkers and MBE, adjusted for age and National Institutes of Health Stroke Scale (NIHSS).

**Results:**

200 patients (69.3 ± 14.3 years; male 55 %) were included for analysis, of whom 26 patients developed MBE (median time from stroke onset to MBE 32.5 h). Compared with patients without MBE, those with MBE had higher level of serum concentration of NLRP3 (median time from onset to blood collection 3 h, 1.85 ng/ml vs. 1.11 ng/ml, *P* = 0.026). NLRP3 level was positively correlated with NIHSS on admission (Spearman ρ = 0.18, *P* = 0.01) and the association between NLRP3 and MBE was attenuated (OR 1.47, 95 % CI 0.88–2.46, *P* = 0.138) after adjusting for age and NIHSS. There was no significant difference in other biomarkers between MBE and non-MBE groups.

**Conclusions:**

There was a trend of association between a higher level of serum concentration of NLRP3 and an increased risk of MBE after ischemic stroke, possibly confounded by the severity of stroke, which is worth further validation in large cohort studies.

## Introduction

Stroke is a leading cause of death and disability in the world [1]. Brain edema is the leading cause of death during acute phase of stroke, [2] which can develop rapidly during the first few days after the onset of stroke, causing occupying effect, herniation and even death, known as malignant brain edema (MBE) [3]. The fatality of MBE can be as high as up to 78 % [4]. Hemicraniectomy decompression is an effective treatment to improve survival after MBE, but it is underused in practice [5, 6]. A phase 2 A clinical trial reported that intravenous glyburide reduced midline shift and edema-related death in patients with large hemispheric infarction, but it did not reduce the risk of MBE nor improve 3-month functional outcomes [7], where its phase 3 trial (NCT02864953) is ongoing. Given the lack of widely-accepted therapies to prevent or treat MBE, it is essential to explore its underlying mechanism to inform the potential intervention target.

Neuroinflammation and related damage of blood-brain barrier (BBB) are possible mechanisms of the development of brain edema following stroke [8]. The rat stroke model showed that systemic inflammation could augment neuroinflammation and aggravate BBB damage and brain edema [9]. Inflammasomes play a key role in neuroinflammatory responses in neurological diseases including ischemic stroke [10, 11]. The nucleotide-binding oligomerization domain-like receptor family pyrin domain-containing 3 (NLRP3) inflammasome is the most commonly studied inflammasome in stroke [12]. In mice stroke model, the expression of NLRP3 inflammasome was increased in ischemic brain tissue and promoted the damage of BBB, possibly through the pathway of pyroptosis [13, 14]. NLRP3 inflammasome can lead to BBB damage through the activation of caspase-1 (CASP1) [15]. and matrix metalloproteinase-9 (MMP9) [16]. In addition, hypoxia inducible factor-1α (HIF-1α) and cathepsin B (CTSB) are increased in the ischemic brain tissue, which are potential activators of NLRP3 inflammasome [17, 18]. Despite promising results from animal studies, the association of NLRP3 and its related inflammatory factors with BBB damage-related complications such as brain edema has not been explored in clinical settings.

Therefore, we performed this study to explore the association of NLRP3 and its related inflammatory biomarkers (HIF-1α, CTSB, CASP1 and MMP9) with MBE in patients with acute ischemic stroke.

## Methods

### Study participants

This study was based on a prospective cohort of patients with acute ischemic stroke (NCT03222024). We screened patients with acute ischemic stroke who had been admitted to the Department of Neurology, West China Hospital, Sichuan University, from Jan 2019 to Sept 2019. Inclusion criteria: (a) admitted within 24 h after stroke onset, (b) anterior circulation infarction confirmed by brain CT/MRI, (c) consented to participate. Exclusion criteria: (a) intracranial hemorrhage or stroke mimics as confirmed by brain CT/MRI, (b) did not have brain CT within 24 h after stroke onset, (c) did not have repeated brain CT/MRI within 7 days after stroke onset, (d) did not provide blood sample on admission. This study was performed according to the Helsinki Declaration and approved by the Biomedical Research Ethics Committee of West China Hospital, Sichuan University (No. 2017[130]), and conformed to local and international ethical criteria. Written informed consent was obtained from all patients or their next of kin.

### Collection of clinical information and follow-up

Demographics (age and sex), medical history (hypertension, diabetes mellitus, hyperlipidemia, atrial fibrillation, valvular heart diseases, prior ischemic stroke and prior intracerebral hemorrhage), current smoking and drinking, body temperature, systolic blood pressure (BP) and diastolic BP were collected on admission. Stroke characteristics were assessed and recorded by trained neurologists: the level of consciousness was assessed using the Glasgow Coma Scale (GCS) [19], stroke severity was assessed using the National Institutes of Health Stroke Scale (NIHSS) [20], and stroke subtype was determined by the Trial of ORG 10,172 in Acute Stroke Treatment (TOAST) classification [21]. Acute treatment including intravenous thrombolysis and intra-arterial interventions were noted. Successful recanalization was defined by modified thrombolysis in cerebral infarction score (mTICI) 2b or 3 on digital subtraction angiography (DSA) immediately after intra-arterial intervention. At 3-month after stroke onset, a researcher blind to in-hospital information performed a telephone-interview for each participant to assess their functional status using modified Rankin scale.

### Serum inflammatory biomarkers evaluation

Venous blood samples were collected in a vacuum blood collection tubes with no EDTA in the Department of Emergency on admission, centrifuged immediately at 3000 rpm for 15 min to isolate the supernatant serum, and the serum was frozen at -80 °C until testing. Serum concentration of NLRP3, HIF-1α, CTSB, CASP1 and MMP9 were analyzed by enzyme-linked immunosorbent assay (ELISA) kits: HIF-1α (E-EL-H6066; Elabscience, Wuhan, Hubei, China), NLRP3 (E-EL-H2557c; Elabscience), CTSB (E-EL-H6004; Elabscience), CASP1 (E-EL-H0016c; Elabscience) and MMP9 (E-EL-H6075; Elabscience) according to the manufacturer’s protocol (https://www.elabscience.cn/). The ELISA kit used the Sandwich-ELISA principle with the microplates pre-coated with antibodies specific to each biomarker. First, the serum was used as the stock solution for CASP1, and diluted 5-folds for NLRP3, HIF-1α and MMP9, and 10-folds for CTSB. Then, 100 ul diluted serum of each sample and the standard sample were added to the micro-ELISA plate wells and incubated at 37 °C for 1.5 h. Second, the reaction system was removed from each well and biotinylated detection antibodies were added and incubated at 37 °C for 1 h. Third, after biotinylated detection antibodies were removed by washing with distilled deionized water for 3 times, avidin-horseradish peroxidase conjugate was added and incubated at 37 °C for 1 h. Next, the substrate solution was added to each well after washing with distilled deionized water for 5 times and incubated at 37 °C for 15 min. Finally, the stop solution was added to each well and the optical density (OD) of each well was measured spectrophotometrically at the wavelength of 450nm.

### Imaging assessment

All patients underwent a brain CT scan on admission and a second brain imaging by CT or MRI < 7 days after stroke onset. For patients with neurological deterioration, an emergency brain CT was performed. Two trained neurologists independently reviewed all brain images to assess the presence of MBE.

### Outcome measures

The primary outcome was MBE, defined as neurological deterioration (depressed level of consciousness and/or increased NIHSS score by 4 points or more, with or without the presence of anisocoria) with imaging signs of midline shift or compressed basal cisterns [22]. Secondary outcomes included (a) death in-hospital or within 7 days of discharge, (b) death at 3 months, (c) poor functional outcome at 3 months (defined as modified Rankin Scale score ≥ 3), and (d) parenchymal hematoma (defined as hemorrhage of brain parenchyma with mass effect according to the European Cooperative Acute Stroke Study II criteria) [23].

### Statistical analysis

Data were reported as mean ± standard deviation (SD) or median (interquartile range, IQR) for continuous variables, or frequencies with percentages for categorical variables. Data were examined for normality using the Kolmogorov-Smirnov test, where the data did not fit the normal distribution, non-parametric analysis was performed with the natural log (Ln)-transformed data. Univariate analysis was performed to explore the association between potential risk factors and each outcome measure. Categorical variables were compared between groups with the Chi-squared test or Fisher’s exact test when appropriate, and continuous variables with the Student’s t test or the Mann-Whitney U test. Comparison between multiple groups was performed by ANOVA. Univariate logistic regression analysis and multivariable logistic regression analysis were performed to assess the association between each inflammatory biomarker and MBE. Multivariable logistic regression was adjusted for the effect of age and NIHSS, which are two important clinical factors associated with MBE [22].. The odds ratio (OR) and 95 % confidence interval (CI) were calculated. Spearman’s coefficients were calculated for the correlation between biomarkers, and between the biomarker and NIHSS as well as body temperature, systolic BP and diastolic BP on admission.

All statistical analysis was conducted using R version 3.6.1 (R Foundation for Statistical Computing, Vienna, Austria) and EmpowerStats (http://www.empowerstats.com, X&Y Solutions, Inc., Boston, MA, USA). A two-tailed *P* < 0.05 was considered statistically significant.

## Results

1159 patients with acute ischemic stroke were admitted between January 2019 and September 2019, of whom 592 were admitted within 24 h after the onset of stroke. 200 patients (69.3 ± 14.3 years; male 55 %) satisfied the inclusion criteria and consented to participate were included for analysis, where 26 (13 %) patients developed MBE (median time from stroke onset to MBE was 32.5 h, IQR 19-53.3 h, range 10.7-82.4 h), 41 (20.5 %) patients presented parenchymal hematoma, and 21 (10.5 %) patients died in-hospital or within 7 days after discharge. Of 195 (97.5 %) patients who completed follow-up interview at 3 months, 41 (21 %) patients died and 94 (48.2 %) patients had poor functional outcome. Of 26 patients with MBE, 16 (61.5 %) were cardioembolic stroke, 9 (34.6 %) were large arterial atherosclerosis, and 1 (3.8 %) was post-surgery for aortic dissection. Patients with history of atrial fibrillation, higher NIHSS score, lower GCS score, or those with depressed level of consciousness on admission were more likely to develop MBE. Intraarterial intervention with successful recanalization reduced the risk of MBE (Table 1).

**Table 1:** The baseline characteristics of stroke patients with or without MBE

	MBE (*n* = 26)	Non-MBE (*n* = 174)	*P*-value
Age (years), mean±SD	72.92 ± 9.19	68.78 ± 14.89	0.17^a^
Male, n (%)	12 (46.15 %)	97 (55.75 %)	0.36^c^
Onset to admission time (hours), median (IQR)	3.00 (1.00–4.00)	3.00 (2.00–4.00)	0.93^d^
Onset to blood sample collection time (hours), median (IQR)	3.00 (1.00-4.31)	3.00 (2.00–4.00)	0.94^d^
Medical history			
Hypertension, n (%)	16 (61.54 %)	96 (55.17 %)	0.54^c^
Diabetes mellitus, n (%)	3 (11.54 %)	40 (22.99 %)	0.19^c^
Hyperlipidemia, n (%)	3 (11.54 %)	12 (6.90 %)	0.40^c^
Atrial fibrillation, n (%)	16 (61.54 %)	70 (40.23 %)	0.04^c^
Valvular heart diseases, n (%)	4 (15.38 %)	35 (20.11 %)	0.57^c^
Prior ischemic stroke, n (%)	4 (15.38 %)	31 (17.82 %)	0.76^c^
Prior intracerebral hemorrhage, n (%)	1 (3.85 %)	8 (4.60 %)	1.00^c^
Current smoking, n (%)	5 (19.23 %)	42 (24.14 %)	0.58^c^
Current drinking, n (%)	2 (7.69 %)	29 (16.67 %)	0.24^c^
Admission body temperature, median (IQR)	36.5 (36.5–36.6)	36.5 (36.3–36.5)	0.11^b^
Admission systolic BP, mean±SD	151 ± 28	144 ± 24	0.15^a^
Admission diastolic BP, mean±SD	84 ± 14	81 ± 13	0.30^a^
TOAST classification			0.16^c^
Large-artery atherosclerosis, n (%)	9 (34.62 %)	44 (25.29 %)	
Small-artery occlusion, n (%)	0 (0.00 %)	22 (12.64 %)	
Cardioembolic, n (%)	16 (61.54 %)	87 (50.00 %)	
Other etiology, n (%)	1 (3.85 %)	8 (4.60 %)	
Undetermined etiology, n (%)	0 (0.00 %)	13 (7.47 %)	
NIHSS on admission, median (IQR)	16 (15–19)	9 (4–16)	< 0.001^b^
NIHSS ≥ 15, n (%)	20 (76.92 %)	60 (34.48 %)	< 0.001^c^
GCS on admission, median (IQR)	13 (8–15)	15 (11–15)	0.02^b^
Disturbance of consciousness on admission	12 (46.15 %)	45 (25.86 %)	0.03^c^
Intravenous thrombolysis, n (%)	8 (30.77 %)	57 (32.76 %)	0.84^c^
Intra-arterial interventions, n (%)	14 (53.85 %)	53 (30.46 %)	0.02^c^
Intra-arterial interventions with successful recanalization	7/14 (50 %)	44/53 (83.02 %)	0.01^c^
Inflammatory biomarkers, median (IQR)			
HIF-1α (pg/ml)	122.50 (84.75-211.38)	142.25 (91.25-238.25)	0.17^b^
CTSB (pg/ml)	3700.50 (3327.62-4620.38)	3895.75 (3216.75-4666.75)	0.36^b^
NLRP3 (ng/ml)	1.85 (0.98–3.87)	1.11 (0.94–2.44)	0.03^b^
CASP1 (pg/ml)	21.25 (8.84–45.29)	19.15 (3.10-48.89)	0.88^b^
MMP9 (ng/ml)	32.27 (24.10-35.36)	30.68 (22.85–35.94)	0.95^b^
Clinical outcomes			
Death in hospital or within 7 days of discharge	14 (53.85 %)	7 (4.02 %)	< 0.001^d^
Loss to follow-up at 3 months	0	5 (2.87 %)	0.38^c^
3-month death	22/26 (84.62 %)	19/169 (11.24 %)	< 0.001^c^
3-month mRS, median (IQR)	6.00 (4.00–6.00)	2.00 (0.00–6.00)	< 0.001^b^

BP: blood pressure; CASP-1, Caspase-1; CTSB, cathepsin B; HIF-1α, hypoxia inducible factor-1α; GCS, Glasgow Coma Scale; IQR, interquartile range; MBE, malignant brain edema; MMP9, Matrix Metalloproteinase-9; mRS, modified Rankin Scale; NIHSS, National Institutes of Health Stroke Scale; NLRP3, nucleotide-binding oligomerization domain-like receptor family pyrin domain-containing 3

a Student’s test, b Mann-Whitney Test, c Chi-squared test, d Fisher’s exact test. Data with non-normal distribution are presented in their original form with p values for non-parametric analysis of their Ln-transformed form

Compared with patients without MBE, those with MBE had higher levels of serum concentration of NLRP3 on admission (median concentration 1.85 [IQR 0.98–3.87] ng/ml vs. 1.11 [0.94–2.44] ng/ml, *P* = 0.026; median time from the onset of stroke to blood sample collection 3 h, IQR 2-4 h). In univariate analysis, a higher level of NLRP3 was associated with a higher risk of MBE (OR 1.72, 95 % CI 1.06–2.79, *P* = 0.03). In addition, NLRP3 concentration was positively correlated with NIHSS scores on admission (Spearman ρ = 0.18, *P* = 0.01) and the association between NLRP3 and MBE was attenuated after adjusting for age and NIHSS scores (OR 1.47, 95 % CI 0.88–2.46, *P* = 0.138) (Table 2). No difference was found in serum concentration of NLRP3 between TOAST subtypes (F = 0.70, *P* = 0.59). There was no association between NLRP3 concentration and body temperature (Spearman ρ=-0.01, *P* = 0.90), systolic BP (Spearman ρ = 0.08, *P* = 0.29) or diastolic BP (Spearman ρ = 0.02, *P* = 0.76) on admission.

**Table 2:** Unadjusted and adjusted odds ratios of all biomarkers for malignant brain edema

	Unadjusted		Adjusted ^a^	
	OR (95 % CI)	*P* value	OR (95 % CI)	*P* value
NLRP3	1.72 (1.06, 2.79)	0.03	1.47 (0.88, 2.46)	0.14
HIF 1α	0.72 (0.37, 1.37)	0.31	0.61 (0.31, 1.20)	0.15
CTSB	1.12 (0.26, 4.95)	0.88	0.80 (0.17, 3.79)	0.78
CASP1	1.14 (0.86, 1.51)	0.36	1.15 (0.86, 1.55)	0.34
MMP9	1.00 (0.97, 1.04)	0.95	1.00 (0.96, 1.04)	0.87

CASP-1, Caspase-1; CI: confidence interval; CTSB, cathepsin B; HIF-1α, hypoxia inducible factor-1α; MMP9, Matrix Metalloproteinase-9; NLRP3, nucleotide-binding oligomerization domain-like receptor family pyrin domain-containing 3; OR, odds ratio. Data of serum level of biomarkers were Ln-transformed for analysis. ^a^ adjusted variables included age and National Institutes of Health Stroke Scale score

The serum concentration of NLRP3 was positively correlated with that of CASP1 (ρ = 0.32, *P* < 0.001) and MMP9 (ρ = 0.31, *P* < 0.001), and negatively correlated with CTSB (ρ=-0.29, *P* < 0.001) and HIF-1α (ρ=-0.22, *P* = 0.001). There was no significant difference in serum concentration levels of HIF-1α, CTSB, CASP1 and MMP9 between MBE and non-MBE groups (Table 1). No significant association was found between any tested biomarkers and parenchymal hematoma, in-hospital death, death or functional outcome at 3 months (all *P* > 0.05).

## Discussion

In this study, MBE was a devastating condition developed at a median of 3 days after the onset of stroke symptoms, due to which more than half of the patients died in hospital. With an aim to inform its prediction, we enrolled patients admitted within 24 h after the onset of stroke and collected their blood samples immediately after admission. We found that a higher level of serum concentration of NLRP3 at baseline was possibly associated with a higher risk of MBE. In addition, NLRP3 concentration was positively correlated with NIHSS score, which may explain the attenuation of the association between NLRP3 and MBE after adjusting for the effect of age and NIHSS. The association between NLRP3 and MBE provides additional information for assessing the risk of MBE in patients, particularly for those whose NIHSS score could not be accurately assessed.

Experiment of mice stroke model showed that NLRP3 was involved in BBB breakdown and the formation of brain edema, [24] but this association has not yet been verified in clinical settings. Our study filled this gap, showing a potential association between NLRP3 and MBE, although possibly confounded by stroke severity. NLRP3 is a key factor in pyroptosis, an inflammatory-mediated programmed cell death [25]. Pyroptosis is activated in cerebral ischemia/reperfusion injury, which activated CASP1 to form Gasdermin D pore and thus cause cell swelling and the release of inflammatory factors [11]. We found that the serum concentration of NLRP3 was positively correlated to CASP1, providing preliminary clinical evidence to the involvement of pyroptosis in ischemic stroke. NLRP3 was also associated with MMP9, an indicator of BBB breakdown [26]. We found that the level of serum NLRP3 was negatively correlated with both CTSB and HIF-1α. This was inconsistent with the findings of animal experiments, where the level of NLRP3 was positively correlated with CTSB and HIF-1α in ischemic brain tissue [27, 28]. However, the relationship between the three remains unclear in patients with acute ischemic stroke. A recent study showed that the level of HIF-1α in cerebrospinal fluid among stroke patients was lower in patients with cognitive impairment than those without cognitive impairment, an impairment possibly related to BBB damage [29]. Therefore, the serum level of CTSB and HIF-1α in stroke patients remains inconclusive and their association with MBE after stroke needs further investigation. In general, our findings indicated that NLRP3-related pyroptosis plays an important role in the process of MBE development. In contrast, we found no evidence of the association between NLRP3 and parenchymal hematoma. This implies a possibility that NLRP3 is more involved in MBE than parenchymal hematoma. Future large cohort studies are warranted to further investigate this potential association between a higher level of serum concentration of NLRP3 and an increased risk of MBE in patients with acute ischemic stroke. Once confirmed, a higher level of NLRP3 at an early stage of stroke may help to identify patients at a high risk for MBE. In addition, interventions to inhibit NLRP3 and/or to break down the pyroptosis may reduce the risk of MBE.

Consistent with previous findings, [30, 31] we found that successful recanalization, but not the administration of intravenous thrombolysis or endovascular treatment, was associated with a lower risk of MBE. This indicates the importance of improving recanalization rate of reperfusion therapies and reducing complications such as MBE. Our study may provide a direction for future research to modulate the NLRP3-associated neuroinflammation in patients receiving reperfusion therapies.

This study has some limitations. First, we were able to analyze a sample of 200 patients who consented to provide blood sample and imaging data, but could not present a consecutive cohort. This design included a relative high proportion of patients with MBE (13 %) compared to the reported 1–10 % in patients with supratentorial ischemic stroke [3] and allowed for the comparison between MBE group and non-MBE group. Second, as suggested by animal study that the level of NLRP3 might be consistently increased within the first 24 h after stroke [24], we enrolled patients within 24 h after onset and examined their baseline NLRP3 level. However, we were only able to collect one blood sample on admission which could not reflect a dynamic profile of these serum biomarkers; therefore, our preliminary findings of the association between NLRP3 and MBE imply a possible mechanism for predicting MBE but the involvement of NLRP3 in the mechanism of MBE need further investigation.

## Conclusions

There was a trend of association between a higher level of serum concentration of NLRP3 and an increased risk of MBE in patients with acute ischemic stroke, possibly confounded by the severity of stroke, which is worth further investigation in large cohort studies.

## Data Availability

The data used in this study are available from the corresponding author upon reasonable request.
